# Expression profile of *amh*/Amh during bi-directional sex change in the protogynous orange-spotted grouper *Epinephelus coioides*

**DOI:** 10.1371/journal.pone.0185864

**Published:** 2017-10-10

**Authors:** Guan-Chung Wu, Hau-Wen Li, Wei-Guan Tey, Chien-Ju Lin, Ching-Fong Chang

**Affiliations:** 1 Department of Aquaculture, National Taiwan Ocean University, Keelung, Taiwan; 2 Center of Excellence for the Oceans, National Taiwan Ocean University, Keelung, Taiwan; National Cheng Kung University, TAIWAN

## Abstract

Gonadal differentiation is tightly regulated by the initial sex determining gene and the downstream sex-related genes in vertebrates. However, sex change in fish can alter the sexual fate from one sex to the other. Chemical-induced maleness in the protogynous orange-spotted grouper is transient, and a reversible sex change occurs after the chemical treatment is withdrawn. We used these characteristics to study Amh signaling during bi-directional sex change in the grouper. We successfully induced the female-to-male sex change by chemical (aromatase inhibitor, AI, or methyltestosterone, MT) treatment. A dormant gonad (a low proliferation rate of early germ cells and no characteristics of both sexes) was found during the transient phase of reversible male-to-female sex change after the withdrawal of chemical administration. Our results showed that *amh* (anti-mullerian hormone) and its receptor *amhr2* (anti-mullerian hormone receptor type 2) were significantly increased in the gonads during the process of female-to-male sex change. Amh is expressed in the Sertoli cells surrounding the type A spermatogonia in the female-to-male grouper. Male-related gene (*dmrt1* and *sox9*) expression was immediately decreased in MT-terminated males during the reversible male-to-female sex change. However, Amh expression was found in the surrounding cells of type A spermatogonia-like cells during the transient phase of reversible male-to-female sex change. This phenomenon is correlated with the dormancy of type A spermatogonia-like cells. Thus, Amh signaling is suggested to play roles in regulating male differentiation during the female-to-male sex change and in inhibiting type-A spermatogonia-like cell proliferation/differentiation during the reversible male-to-female sex change. We suggest that Amh signaling might play dual roles during bi-directional sex change in grouper.

## Introduction

In mammals (mice), sex determination results from the initial switch of either the Sry-dependent, testis-differentiating or Sry-independent, ovary-differentiating molecular cascade in an exclusive manner [1]. However, the initiation of both pathways in the same gonad will result in the development of an ovotestis [2]. These ovotestes can be caused by an insufficient or delayed expression of the testis-related gene in the XY gonad [3–5] or loss of the ovary-related gene in the XX gonad [6]. Thus, gonadal differentiation is tightly regulated by the initial sex-determining gene and downstream sex-related genes in mammals.

Unlike most vertebrates that have stable sexes, 2% of fish exhibit hermaphroditism [7, 8]. Many hermaphroditic fish can alter the sexual fate from one sex to the other sex, including protogyny (female-to-male sex change), protandry (male-to-female sex change), and serial sex change (bi-directional sex change) [9]. Sex determination in fish is very sensitive to the endogenous levels of estrogen [10, 11]. In most fish, the blockade of Cyp19a1a activity by the aromatase inhibitor (AI) and induction of estradiol (E2) result in masculinization and feminization, respectively [9, 10]. However, the estrogen-induced femaleness in protandrous black porgy (*Acanthopagrus schlegelii*) [12, 13] and AI-induced maleness in the protogynous orange-spotted grouper (*Epinephelus coioides*) [14] are transient, and a reversible sex change occurs after the chemical treatment is withdrawn. Therefore, the sexual phase is tightly regulated by the endogenous factors of the hermaphroditic fish (age in black porgy and body size in grouper). However, the key gene to switch controlling the gonadal sex differentiation cascade leading to the development of one sex but the regression of the other sex remains unclear in hermaphroditic fish.

Anti-mullerian hormone (AMH), also known as mullerian-inhibitory substance, is the gonadal hormone responsible for the regression of Mullerian ducts in male fetuses during mammalian embryogenesis. AMH signals through the type II AMH receptor (AMHR2) to regulate the differentiation and growth of target cells in mammals [15]. Three different type I AMH receptors (activin-receptor kinase, ALK) are thought to mediate AMH stimulatory (ALK2 and ALK3) and inhibitory (ALK6) response in target cells through AMHR2 binding [16]. Teleost fish lack mullerian ducts, but *amh* homologues and its receptors (*amhr2*) have been identified [17]. In fish, several genes in the Amh signaling pathway are involved in sex determination, including a Y-linked duplicate of *amh* (*amhy*) in Patagonian pejerry (*Odontesthes hatcheri*) [18] and Nile tilapia (*Oreochromis niloticus*) [19] and a mutation in *amhr2* in pufferfish (*Takifugu rubripes*) [20]. Moreover, *amh* is highly expressed in male gonads and is involved in testicular differentiation in fish [17]. In the Japanese eel (*Anguilla japonica*) [21], medaka (*Oryzias latipes*) [22], zebrafish (*Danio rerio*) [23], and black porgy [24], recombinant Amh reduced type A spermatogonia proliferation and spermatogenesis. Furthermore, *hotei* mutation (*amhr2* mutation) results in a male-to-female sex change in XY medaka [25]. Therefore, these findings suggest an important role of Amh signaling in gonadal differentiation in teleosts.

Grouper is a protogynous fish that is important for aquaculture. Sex change is tightly controlled by endogenous factors, such as body size [26]. The exogenous administration of chemicals (AI or methyltestosterone, MT) induce transient maleness (passive maleness), and then a reversible sex change was found after the chemical treatment was withdrawn [14, 27]. In our recent work, a dormant (low proliferation rate of early germ cells) gonad was found in the transient phase during reversible male-to-female sex change in AI- and MT-terminated fish [14]. We used this chemical-induced (bi-directional) sex change to understand the role of Amh signaling in gonadal differentiation and sexual phase maintenance in the orange-spotted grouper. In this study, we found that *amh* and *amhr2* were mainly expressed in the gonad during female-to-male sex change and testes. Amh was localized in the Sertoli cells surrounding the type A spermatogonia-like cells. Amh expression was found in the surrounding cells of type A spermatogonia-like cells during the transient phase of reversible male-to-female sex change. Our results indicate that Amh signaling might regulate male differentiation during female-to-male sex change and prevent advanced differentiation in early germ cells during reversible male-to-female sex change. Therefore, we suggest that Amh plays important roles during bi-directional sex change in the grouper.

## Materials and methods

### Experimental fish

The juvenile mono-female grouper is an advantageous model animal used to investigate the regulation of sexual fate. Juvenile groupers were purchased from the hatchery in Pingtung, Taiwan. The fish were acclimated to the large seawater tank (2.5 tons) at the National Taiwan Ocean University culture station and with a natural lighting system. All procedures and investigations were approved by the National Taiwan Ocean University Institutional Animal Care and Use Committee (approved number 102009) and were performed in accordance with standard guide principles. The fish were anesthetized in 2-phenoxyethanol (0.5 ml/l water) during handling and sampling.

### Induction of bi-directional sex change

**Experiment 1:** To enhance the process of female-to-male sex change, we conducted the sex change by applying MT implantation (S1A Fig). The biological parameters and sexual phase of the gonads were shown in the S1 Table. The fish (>2.5-years-old) were implanted with a methyltestosterone (MT, Kingyoker, Taipei City, Taiwan) pellet (100 μg MT/mg pellet; 200 mg pellet/kg of body weight, n = 18) or control vehicle (n = 24) in the muscle. Approximately all control fish (23/24) showed femaleness during the experimental period. One fish (1/24) was primary male in the control fish. However, no significant size difference was found in this male (body length: 31.8 cm; body weight: 397.4 g) compare with the other females (body length: 31.9 ± 0.5 cm; body weight: 396.1 ± 26.3 g). All fish showed maleness in MT-implanted fish after 4 weeks of administration. The primary oocyte stage and vitellogenic oocyte stage were found in the control fish (S1 Table). A regressed oocyte stage (the initial phase of sex change) and a terminal phase of sex change were found in the MT-implanted fish during the experimental period (1 week, 2 weeks, and 4 weeks after MT implantation) (S1 Table).

**Experiment 2:** To obtain fish in the different sexual phases of bi-directional sex change, we induced chemical-induced sex change by treatment with an aromatase inhibitor (AI: 1,4,6-androstatriene-3,17-dione, ATD, 20 mg/kg of feed, Steraloids, Newport, RI, USA) and MT (50 mg/kg of feed) (S1B Fig). The biological parameters and sexual phase of the gonads were shown in the S2 Table. Juvenile fish (7 months old) were fed a diet with AI or MT twice daily for 12 weeks of chemical treatment. Control fish were fed a control diet without chemicals. All control fish showed femaleness during the experimental period (S2 Table). All fish showed maleness in AI-treated and MT-treated fish after 12 weeks of administration (S2 Table). AI-feeding and MT-feeding were terminated after 12 weeks of oral administration, and all fish in both groups showed altered sexual fate from maleness to femaleness. Testicular tissue had completely degenerated, and an ovary with primary oocytes was found after 9 weeks of AI or MT withdrawal (S2 Table).

### Gonadal histology

The fish gonads were fixed with 4% paraformaldehyde in PBS at 4°C for 16 hours. The fixed gonads were dehydrated in ethanol and then were embedded in paraffin. The gonadal sections (6-μm thickness) were rehydrated and subjected to hematoxylin and eosin staining. The gonads of all of the fish were histologically examined.

### Gene expression profile analysis

The gonadal status of all fish was determined by histology. Total RNA was extracted from the gonad by TRIzol reagent (Invitrogen, Carlsbad, CA, USA). The integrity of total RNA was confirmed by agarose gels. Total RNA run on a denaturing gel had the sharp, clear 28S and 18S rRNA bands in testis. In spite of these 2 bands, a sharp and smaller rRNA band was observed in ovary. The first-strand cDNA was synthesized by Superscript III (Invitrogen) with oligo (dT) 15 primer (Promega, Madison, WI, USA). First-strand cDNA was used for quantitative real-time PCR analysis (qPCR) as previously described [24]. The sequences of *amh* (S2 Fig) and *amhr2* (S3 Fig) were identified using the alignment to compare with other fish species. The specific primers for *dmrt1* (*doublesex and mab-3-related transcription factor 1*, GenBank accession no. EF017802), *sox9* (*sex determining region y-box 9*, GenBank accession no. GQ232762), *cyp11b2* (*11beta-hydroxylase*, GenBank accession no. JQ178340), *amh* (*anti-mullerian hormone*, GenBank accession no. KP161068), *amhr2* (*type II anti-mullerian hormone receptor*, GenBank accession no. KP161069), and *cyp19a1a* (*aromatase gonad form*, GenBank accession no. AY510711) are listed in Table 1. Gene quantification of standards, samples, and controls was conducted simultaneously using qPCR (GeneAmp 7500 Sequence Detection System; Applied Biosystems, Foster City, CA, USA) with SYBR green Master Mix (Applied Biosystems, Vilnius, Lithuania). The PCR specificity was confirmed by a single melting curve (at same temperature) in unknown samples and standards. The respective standard curve of log (transcript concentrations) vs CT (the calculated fractional cycle number at which the PCR-fluorescence product is detectable above a threshold) was obtained. The values detected from different amounts of plasmid DNA containing the fragment of the target gene (10 times of series dilution) of the representative samples were parallel to the respective standard curve. The correlation of the standard curve for the gene analyses was at least -0.999. qPCR assay was conducted with duplicate repeats (n = 6–8 in each group). All samples were normalized to *glyceraldehyde-3-phosphate dehydrogenase* (*gapdh*), and the highest value (control value) of each gene was defined as 100. All data were expressed as the means ± SEM. The values were subjected to analysis via one-way ANOVA, followed by a Student-Newman-Keuls multiple test with *P* < 0.05 indicating a significant difference. Student’s t-test was also conducted to compare the significant differences (*P* < 0.05) between the treatments.

**Table 1:** Oligonucleotides for specific primers.

Gene	Orientation	Sequence
*dmrt1*	Sense	5’-GGCCCTGAGGTGATGGTGAA-3’
	Antisense	5’-CGGGGATCGTCTCTCCACAG-3’
*sox9*	Sense	5’-GGGGCCTTACTGTGTTGC-3’
	Antisense	5’-GCTCCAGAGGCACCAATG-3’
*cyp11b2*	Sense	5’-AGACACGGCAGCACAGCAAG-3’
	Antisense	5’-CAGCCGCACTCATCATCACC-3’
*cyp19a1a*	Sense	5’-CACCAGAGGCACAAGACAGC-3’
	Antisense	5’-CCTGCTCCATGTCTCTCCTC-3’
*amh*	Sense	5’-TTGTGGACATCTTCTCCTGT-3’
	Antisense	5’-CACCAATGAGGATGTCTTTT-3’
*amhr2*	Sense	5’-GAGATCCTGGAGGGCTCTGT-3’
	Antisense	5’-CCCAGCTCAGACTCGTAAGG-3’
*gapdh*	Sense	5’-CGACCCTCACTCCTCCATCTT-3’
	Antisense	5’-GCTGTAGCCGAACTCGTTGTC-3’

### Antibody production

The Amh antiserum was produced in white rabbits immunized against a C-terminal peptide fragment (RATRAGPNNPARGNLC, amino acid 411–427) of the orange spotted grouper Amh (S2 Fig). This peptide fragment was located on the predicted proteolytic cleavage site (RGLRATR, amino acid 408–414) (S2 Fig). The peptide fragment was conjugated with KLH to immunize the antiserum. The antisera were prepared by Kelowna International Scientific, Inc. The specificity of the antiserum was confirmed by Western blot (WB) analysis in this study. WB was performed as our previously described [28]. Anti-actin antibody (1:10000 dilution; product no. MAB1501; Merk Millipore, Billerica, MA, USA) was used to detect actin as the internal control of Amh expression normalization. Immunoblotting was performed using preabsorbed antibodies at 4°C for overnight. Finally, the BCIP/NBT Liquid Substrate System (Sigma, St. Louis,MO, USA) was used to detect protein staining.

### Immunohistochemical staining and immunofluorescent staining

IF (immunofluorescence) and IHC (immunohistochemical) staining were performed as previously described [28]. The fish gonads were fixed with 4% paraformaldehyde in PBS at 4°C for 16 hours. The rehydrated slides were treated with HistoVT One (Nacalai tesque, Kyoto, Japan) to expose the antigens of the target protein. Anti-Vasa (1:1000 dilution; our own antibody) [28], anti-Proliferating cell nuclear antigen (Pcna; 1:250 dilution; product no. sc-7907; Santa Cruz Biotechnology, Billerica, MA, USA), anti-Brdu (1:1000 dilution; product no. MAB4072; Merk Millipore), and anti-Amh (1:1000; present study) were used for IHC and IF staining. For IHC staining, each section was rehydrated in PBS and incubated with 3% H_2_O_2_ in PBS. The section was then incubated with 5% nonfat milk powder for 30 min with antibody overnight at 4°C. This was followed by incubation with an appropriate biotinylated antibody (Vector Laboratories Inc., Burlingame, CA). Color formation was amplified with an ABC kit (avidin-biotin, Vector, Burlingame, CA, USA) and DAB (3,3’-diaminobenzidine, Sigma). For IF staining, the section was then incubated with 5% nonfat milk powder for 30 min with antibody overnight at 4°C. Alexa Fluor secondary antibodies (Invitrogen, Carlsbad, CA, USA) were used. All staining was conducted with triplicate sections for each tissue (n > 3 fish in each group).

### Cell proliferating assay

Brdu incorporation into gonadal cells was used to analyze the proliferating activities. To examine the fate of male germ cells, Brdu (Sigma) was used to label the dividing cells after AI- and MT withdrawal in experiment 2. The fish were injected (intraperitoneal injection; i.p.) with Brdu (0.3 mg/g of body weight) on day 7 and day 4 before sampling. Anti-Brdu (1:1000 dilution; product no. MAB4072; Merk Millipore) was used for IHC to identify the proliferating cells during the treatment period.

## Results

### Stable female phase with unstable male phase in younger and smaller fish

**Fig 1:**
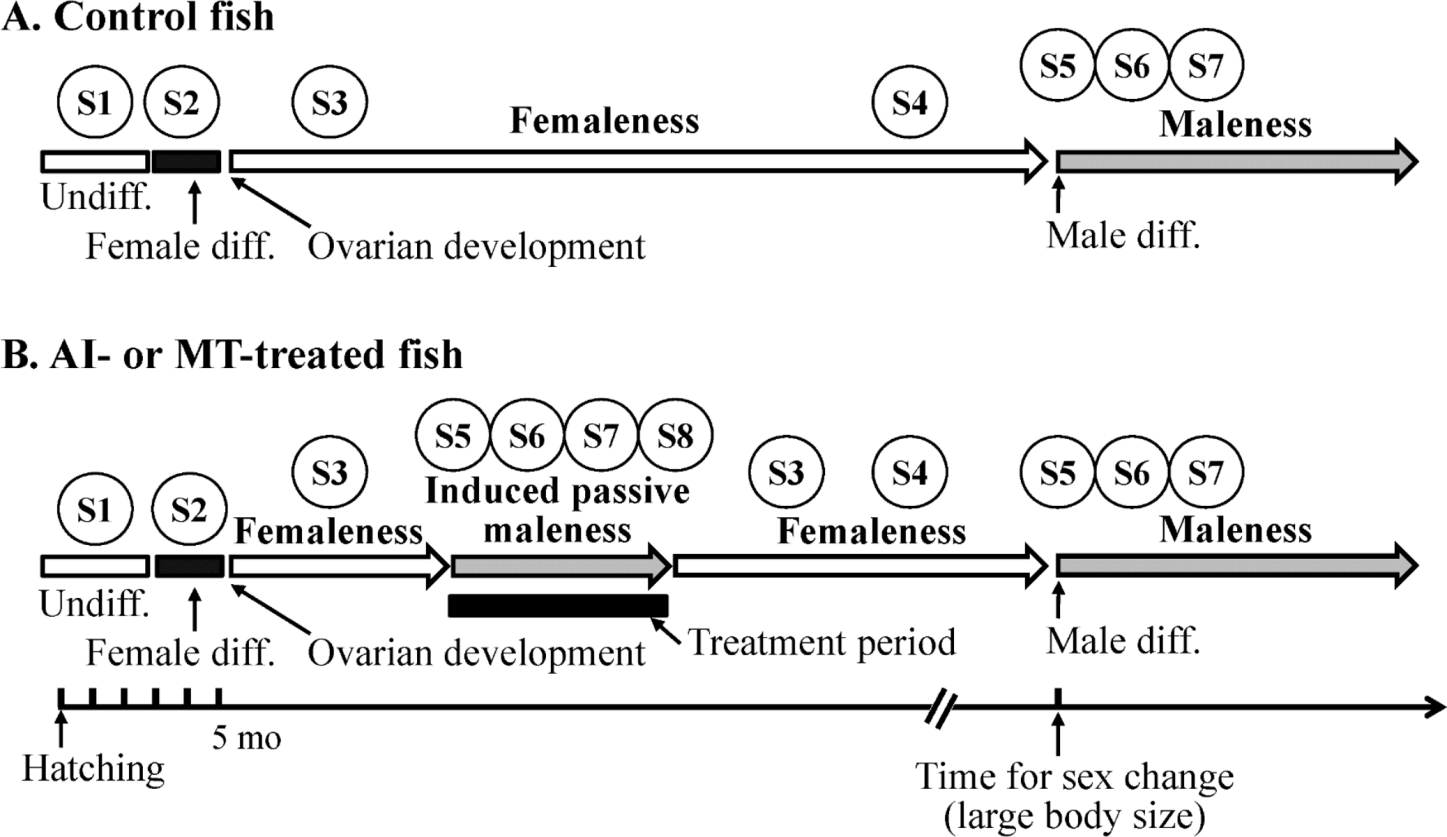
Profiles of gonadal development in control fish and chemical-induced sex change. (A) Control fish: Approximately 100% juvenile fish gonads were differentiated to ovary and were maintained in femaleness. Fish were altered in the sexual phase from femaleness to maleness until they reached a large body size. (B) AI (aromatase inhibitor)- or MT (methyltestosterone)-treated fish: After a few months of AI or MT administration, the ovary was completely regressed, and a functional male was found (female-to-male sex change). This AI- or MT-induced maleness was transient (passive maleness), and the reversible sex change (from male-to-female) was found after AI or MT withdrawal. Eight different stages were classified during bi-directioanl sex change, including status 1 (undifferentiated gonad), status 2 (differentiated ovary), status 3 (primary oocyte stage in femaleness), status 4 (vitrellogenic oocyte stage in femaleness), status 5 (transition phase of female-to-male sex change), status 6 (initial phase of female-to-male sex change), status 7 (terminal phase of female-to-male sex change), and status 8 (transition phase of male-to-female sex reversal). S, status; Undiff, undifferentiated gonad; Diff, differentiation.

Fig 1 is a schematic picture of gonadal development in different conditions, including body size and chemicals treatment (Fig 1A and 1B). Based on histological characteristics, 8 different stages were classified in this study. Fish had an undifferentiated gonad (status 1) and lumen structure was observed after gonadal differentiation during 3-to-4-mo-old fishes (status 2). All juvenile fish entered the female phase. Femaleness fish contained the primary oocyte (status 3) in immature fish and the vitellogenic oocyte (status 4) in mature fish. In the transition phase of female-to-male sex change, many regressed oocytes were observed in the gonad (status 5). In the initial phase of female-to-male sex change, large number of aggregated somatic cells was observed in the gonad (status 6). In the terminal phase of female-to-male sex change, male characteristics were observed in functional male (status 7). After long-term AI or MT administration, precocious males were observed in younger and smaller fish. However, this induced maleness was unstable and reversal sex change was observed after chemical withdrawal (also called passive maleness). In the transition phase of male-to-female sex reversal, dormant early germ cells were observed in the gonad (status 8). Thus, the gonadal stage in grouper was divided into 3 different sexual categories (Fig 1), namely “femaleness”, “maleness” (natural sex change fish), and induced “passive maleness” (AI- or MT-treated fish).

### *amh* and *amhr2* were mainly expressed in the testes

**Fig 2:**
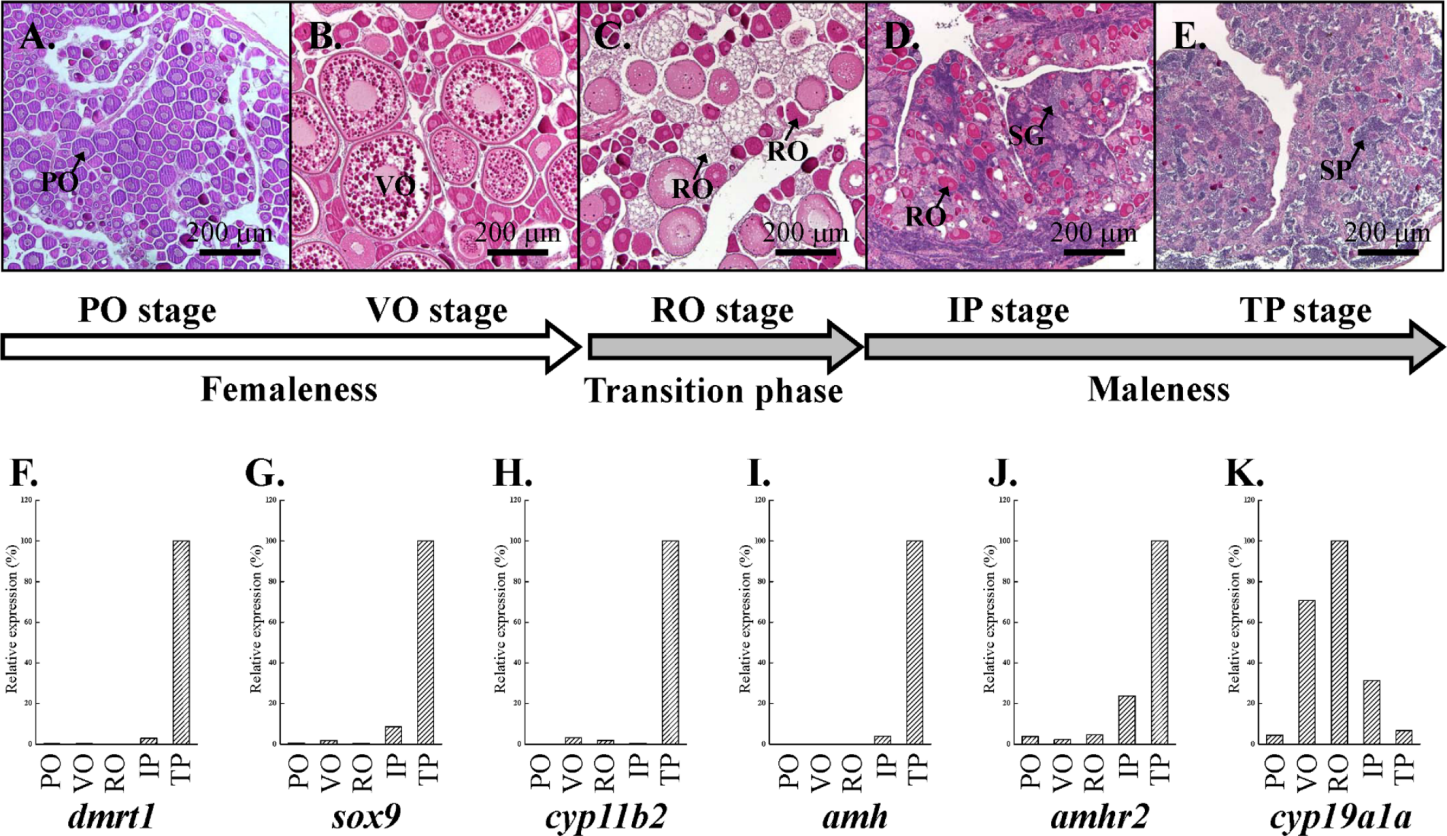
Male-related gene expression profiles during female-to-male sex change. (A-E) According to the histological events, 5 different stages were used for the gene expression pattern in femaleness (A and B), transition phase (C), and maleness (D and E). (A) Primary oocyte stage. (B) Vitellogenic oocyte stage. (C) Regressed oocyte stage. (D) Initial phase of the female-to-male sex change. (E) Terminal phase of the female-to-male sex change. (F-K) Three different categories were used to analyze the gene expression profiles, including the male germ cell marker, *dmrt1* (F) and Sertoli cell marker, *sox9* (G); key enzyme for 11-KT synthesis, *cyp11b2* (H); *amh* (I); *amhr2* (J); and enzyme for E2 synthesis, *cyp19a1a* (K). IP, initial phase; PO, primary oocyte; RO, regressed oocyte; SG, spermatogonia; SP, sperm; TP, terminal phase; VO, vitellogenic oocyte.

In experiment 1, we conducted the sex change by applying MT implantation. The primary oocyte stage (status 3) and vitellogenic oocyte stage (status 4) were found in the control fish (S1 Table). A transition phase of sex change (status 5), an initial phase of sex change (status 6) and a terminal phase of sex change (status 7) were found in the MT-implanted fish during the experimental period (S1 Table). Histological data showed that the gonads of femaleness fish at the primary oocyte stage (PO stage, status 3, Fig 2A) and vitellogenic oocyte stage (VO stage, status 4, Fig 2B). Regressed oocytes were observed in the transition phase during sex change, while there was no sign of testicular differentiation (RO stage, status 5, Fig 2C). Some regressed oocytes with spermatogonia were observed in the initial phase of maleness (IP stage, status 6, Fig 2D). In the terminal phase of maleness, the ovarian tissue had completely degenerated, and sperm was produced in testes with male function (TP stage, status 7, Fig 2E).According to the qPCR results, high expression of *dmrt1* (a male germ cell marker, [29], *sox9* (a Sertoli cell marker, [29], and *cyp11b2* (a key enzyme for 11-KT synthesis, [30] in the TP stage confirmed the histological results (Fig 2F–2H). *amh* and *amhr2* were expressed exclusively in the TP stage of maleness (Fig 2I and 2J). Conversely, high *cyp19a1a* expression was observed in the VO and RO stages (Fig 2K). Thus, *amh* and *amhr2* were highly expressed in the testes, and they showed sex-dimorphic expression.

### Amh was localized in the Sertoli cells surrounding the spermatogonia

**Fig 3:**
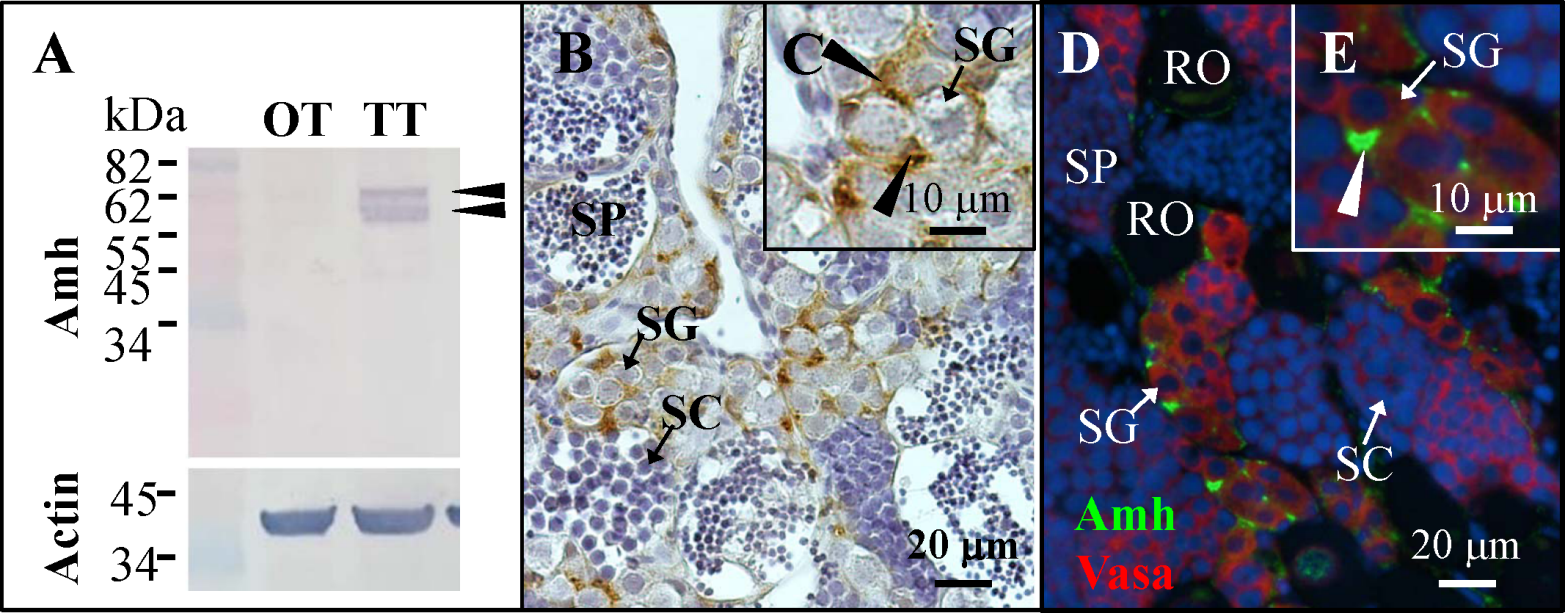
Location of Amh in the testes. (A) Western blotting of grouper testicular and ovarian protein extracts using anti-Amh antiserum and anti-Actin antiserum. The black arrowhead denotes Amh protein. (B and C) Immunohistochemical staining of testes using anti-Amh antiserum. Black arrowheads indicate the positive signal (brown color) of anti-Amh antiserum. (D and E) Immunofluorescent staining of testes using anti-Amh antiserum and anti-Vasa antiserum. Vasa is a germ cell marker. White arrowheads indicate a positive signal (green color) of anti-Amh antiserum. OT, ovarian tissue; RO, regressed oocyte, SC, spermatocyte; SG, spermatogonia, SP, sperm; TT, testicular tissue.

The specificity of Amh antibody was confirmed by a Western blot (WB) analysis using ovary and testis extracts. Actin was used as a reference for WB analysis. Grouper Amh is synthesized as a 518-amino acid pre-pro-protein (pre-pro-Amh). Amh includes an N-terminal leader sequence (MLFVDIFSCGALMLCCARLCAA, amino acid 1–22) and 2 predicted plasmin protease cleavage sites R-X-X-R (S2 Fig). The pro-Amh (after cleavage of signal peptide) had a theoretical size of 53.4-kDa based on the ExPASy website (http://web.expasy.org/compute_pi/). Compared with other fish species (black porgy, European sea bass, and zebrafish), the data showed that RXXRXXR (RGLRATR, amino acid 408–414 in grouper) was conserved in all fishes, but not the RXXR (RLGR, amino acid 365–368 in grouper) (S2 Fig). The pro-Amh contained a 41.4-kDa NH2-terminal fragment with a short peptide of “RGLRATR” (the recognition site of enzymatic cleavage) and a 12-kDa C-terminal fragment (S4 Fig). Furthermore, 3 glycosylation sites were predicted on the grouper pro-Amh based on the NetOGlyc 4.0 Server (http://www.cbs.dtu.dk/services/NetOGlyc/). According to our data, immunoblots of testes (status 7) protein extracts revealed that two Amh proteins of 58-kDa and 68-kDa were clearly detected using the anti-Amh antibody (Fig 3A). No immunoblot signal of Amh was observed in ovary (status 4) protein extracts (Fig 3A). These detected grouper Amh proteins were bigger than the theoretical size (53.4-kDa). These unexpected/alternative processings of grouper Amh in comparison to mammalian Amh were also observed in zebrafish (two candidate full-length Amh proteins of 66 and 71 kDa) [23], black porgy (two candidate full-length Amh proteins of 62 and 55 kDa) [24] and European sea bass (two candidate full-length Amh proteins of 68 and 58 kDa) [31] (S4 Fig). In zebrafish, *in vitro* plasmin-treated Amh shows the different cleavage pattern (more than one cleavage site) compared with human AMH [23]. However, this size discrepancy of endogenous Amh between the observed and theoretical molecular mass remains unclear in fish. In contrast, C-terminal fragment of Amh was not observed in WB (Fig 3A). These results may indicate that the epitope site of the antigenic peptide fragment (RATRAGPNNPARGNLC) may be part of the fragment of “RGLRATR” (Fig 3A). Thus, Amh antibody can’t recognize the N-terminal and C-terminal Amh fragment after Amh processing through the potential plasmin protease cleavage site (RGLRATR). Furthermore, these results also reveal that plasmin protease cleavage site (RGLRATR, amino acid 408–414 in grouper) upstream of the Tgfb-domain that may be necessary for the processing of the grouper Amh. Grouper Amh antibody only identified the pro-Amh but not N-terminal and C-terminal fragment of processed Amh. Taken together, our WB result confirmed that *amh*/Amh expression was mainly in testes but not in ovary. Using immunofluorescence (IF) and immunohistochemical (IHC) staining, we examined the cellular localization of Amh using a specific anti-Amh antibody. IHC staining showed that Amh was found in the Sertoli cells surrounding the spermatogonia, and no signal was observed in the germ line cells (Fig 3B and 3C). Furthermore, we used the anti-Vasa antibody, a germ cell marker in the grouper [29], to confirm the localization of Amh in Sertoli cells by IF staining. IF staining confirmed that Amh signals were expressed in Sertoli cells surrounding the spermatogonia (Fig 3D and 3E).

### Increased Amh expression during the female to male sex change

**Fig 4:**
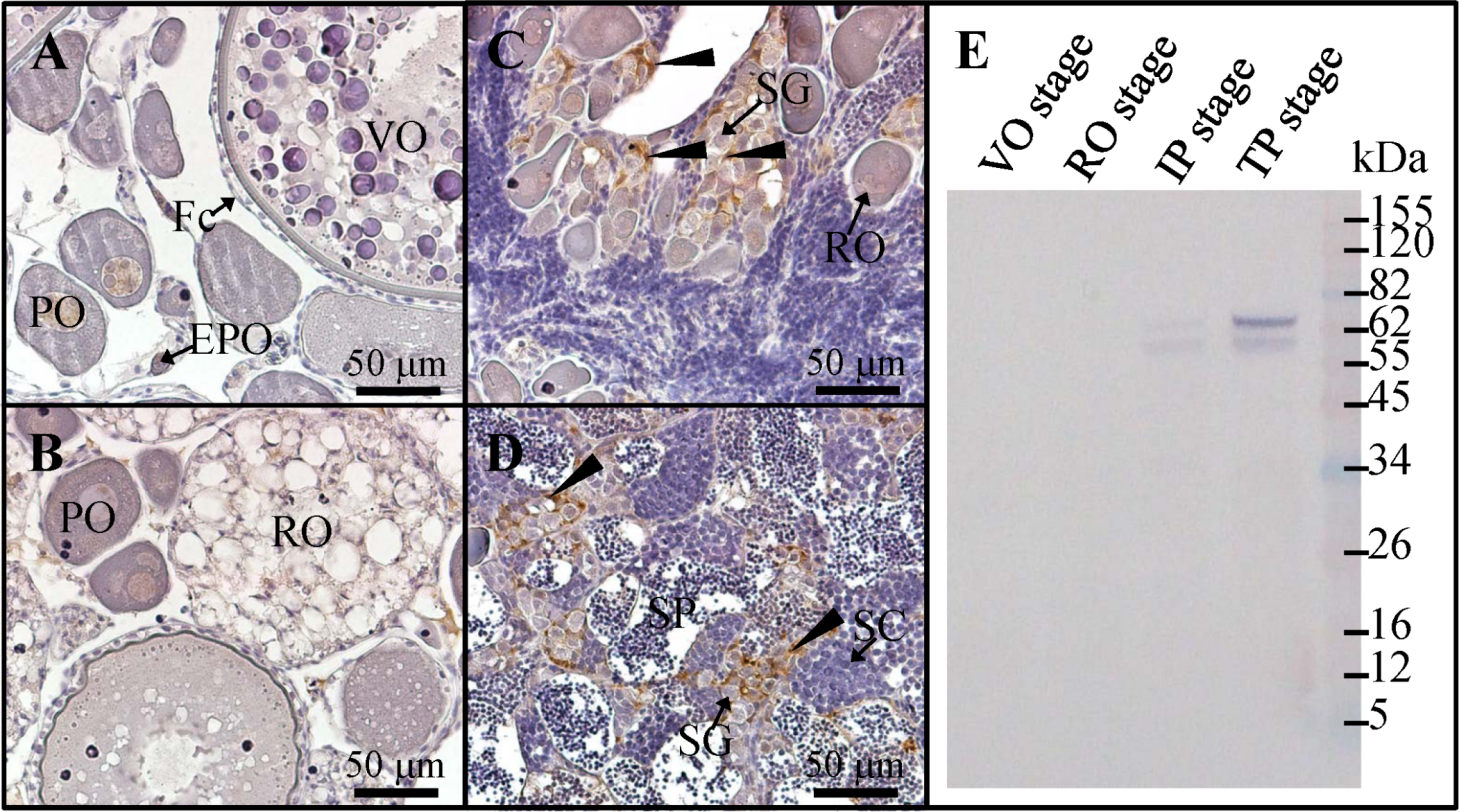
Immunohistochemical analysis of Amh in the gonad during the female-to-male sex change. Immunohistochemical staining of gonads at different stages using anti-Amh antiserum. (A) Vitellogenic oocyte stage. (B) Regressed oocyte stage. (C) Initial phase of the female-to-male sex change. (D) Terminal phase of the female-to-male sex change. (E) Western blotting of grouper gonad protein extracts using anti-Amh antiserum. Black arrowheads indicate the positive signal (brown color) of anti-Amh antiserum. EPO, early primary oocyte; Fc, follicle cell; IP, initial phase; PO, primary oocyte; RO, regressed oocyte; VO, vitellogenic oocyte; SC, spermatocyte; SG, spermatogonia; SP, sperm; TP, terminal phase.

In experiment 1, we conducted the sex change by applying MT implantation. IHC staining revealed that Amh signals were low or absent in the ovary, including early primary oocytes, primary oocytes, vitellogenic oocytes, and follicle cells (Fig 4A). Slight or absent Amh signals were observed in the follicle cells of regressed oocytes in the RO stage (Fig 4B). These regressed oocyte-associated follicle cells did not express Amh in the IP stage (Fig 4C). By contrast, Amh signals were high in Sertoli cells surrounding the spermatogonia in the IP (Fig 4C) and TP stages (Fig 4D). Furthermore, WB data further confirmed our IHC results (Fig 4). Amh signals were observed in the initial phase (with faint Amh signals) and terminal phase (with strong Amh signals) of the female-to-male sex change (Fig 4E).

### Transient maleness in AI- and MT-treated fish

**Fig 5:**
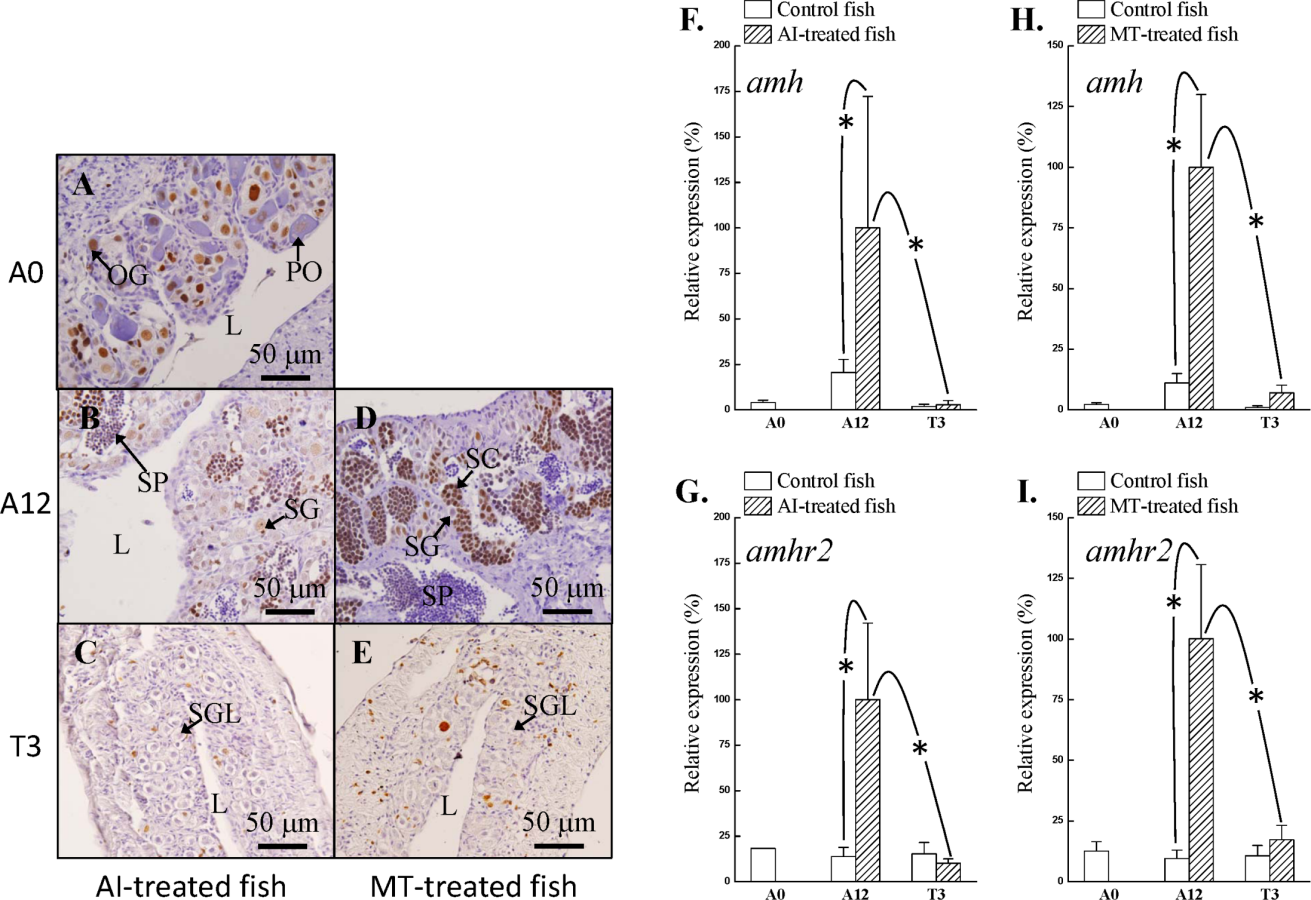
*amh* and *amhr2* expression profiles during bi-directional sex change. Immunohistochemical staining of gonads using anti-Pcna antiserum (A, B, and D) and anti-Brdu antiserum (C and E). Pcna as a proliferating marker and Brdu-incorporated cells revealed high proliferating activity. (A) Ovary at 0 day before AI or MT administration. (B) Testes at 12 weeks after AI administration. (C) Regressed testes at 3 weeks after AI withdrawal. (D) Testes at 12 weeks after MT administration. (E) Regressed testes at 3 weeks after MT withdrawal. *amh* and *amhr2* expression profiles in different statuses in AI-treated fish (F and G) and MT-treated fish (H and I). Asterisk indicates a Student’s *t*-test (*P* < 0.05). Student’s *t*-test was also conducted to compare the significant differences (*P* < 0.05) between the treatments. L, central lumen; OG, oogonia; PO, primary oocyte; SG, spermatogonia; SGL, type A spermatogonia-like cell; SP, sperm.

We induced chemical-induced sex change by treatment with an aromatase inhibitor (AI: 1,4,6-androstatriene-3,17-dione, ATD, 20 mg/kg of feed) and MT (50 mg/kg of feed) in experiment 2. The primary oocyte stage (status 3) was found in the control fish (S2 Table). A transition phase of sex change (status 5), an initial phase of sex change (status 6) and a terminal phase of sex change (status 7) were found in the MT-implanted fish during the experimental period (S2 Table). A dormant stage of gonad (status 8) was obtained during male-to-female reversal sex change (S2 Table). Juvenile females (7-months-old fish with oogonia and primary oocytes, status 3) were used for the AI/MT-induced maleness (Fig 5A). IHC staining of Pcna (proliferating marker) revealed that oogonia had high proliferation activity in juvenile females (Fig 5A). The ovarian tissue had completely degraded and was changed into testicular tissue (status 7) after 12 weeks of AI administration (Fig 5B). Spermatogonia with a small area of advanced male germ cells (including spermatocytes and spermatozoa) were observed in AI-treated fish (Fig 5B). Pcna staining revealed that male germ cells had high proliferation activities in AI-treated fish (Fig 5B). However, this AI-induced maleness has a transient status, and male characteristics were diminished after AI withdrawal, and then a reversible sex change (from male to female) occurred. A dormant stage of the gonad (status 8) was found during the transient phase of male-to-female sex change after the chemical was withdrawn. Type A spermatogonia-like cells were found in the gonad (the transient and dormant stage) after 3 weeks of AI withdrawal (Fig 5C). To examine the fate of male germ cells, Brdu was used to label the divided cells after AI withdrawal. No Brdu-incorporated germ cells were found in this dormant stage of the gonad after 3 weeks of AI withdrawal (Fig 5C).

Similar to the AI-treated fish, the ovarian tissue was almost completely degraded and changed into testicular tissue after 12 weeks of MT administration (Fig 5D). A reversible sex change from passive maleness to femaleness occurred after MT withdrawal. Pcna staining revealed that male germ cells had high proliferation activity in MT-treated fish (Fig 5D). However, only the type A spermatogonia-like cells were found in the gonad after 3 weeks of MT withdrawal (Fig 5E). Few type A spermatogonia-like cells showed Brdu incorporation after 3 weeks of MT withdrawal (Fig 5E). Thus, a dormant status of the transient gonad was found in AI- or MT-treated fish after chemical withdrawal.

### Immediate sexual fate alternation in AI- or MT-treated fish after chemical withdrawal

According to qPCR analysis, our results showed high *amh* (Fig 5F) and *amhr2* (Fig 5G) expression in AI-treated fish compared with that in control fish. The expression levels of *amh* (Fig 5F) and *amhr2* (Fig 5G) were significantly decreased and reduced to control fish levels after 3 weeks of AI withdrawal. Similar to AI-treated fish, high *amh* (Fig 5H) and *amhr2* (Fig 5I) expression was found in MT-treated fish compared with that in control fish. The expression of *amh* (Fig 5H) and *amhr2* (Fig 5I) was significantly decreased and reduced to control fish levels after 3 weeks of MT withdrawal.

**Fig 6:**
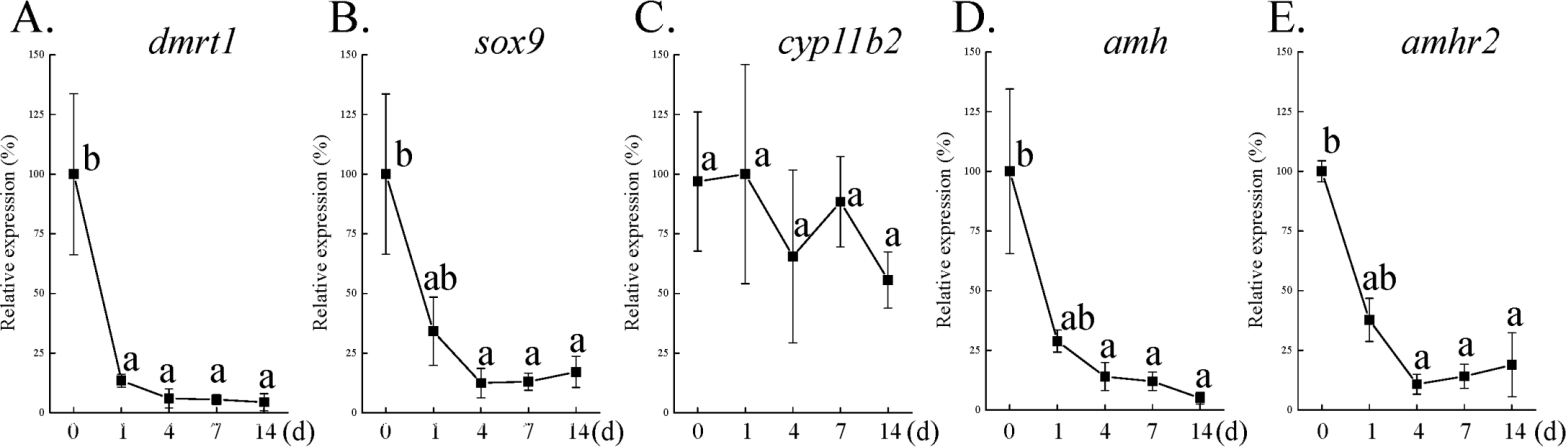
Male-related gene expression profiles during reversible male-to-female sex change. MT-induced maleness is a transient status. Male characteristics were diminished after methyltestosterone (MT) withdrawal (1–2 weeks), and then a male-to-female sex change occurred. Expression of male-related genes *dmrt1* (A), *sox9* (B), *cyp11b2* (C), *amh* (D), and *amhr2* (E) during the initial phase of male-to-female sex change in MT-terminated fish (day 0 to day 14). Superscript letters indicate one-way ANOVA and Student-Newman-Keuls multiple test (*P* < 0.05).

To further investigate the period of this immediately sexual fate alternation in MT-terminated fish, qPCR data were used to analyze the expression profiles of male-related genes, including *dmrt1*, *sox9*, *cyp11b2*, *amh*, and *amhr2*. qPCR data showed that *dmrt1* (male germ cell marker [29]) and *sox9* (Sertoli cell marker [29]) expression (Fig 6A and 6B) was significantly decreased after 1 day and 4 days of MT withdrawal, respectively. No change was found in *cyp11b2* (key enzyme for 11-KT synthesis [30]) expression after 1–14 days of MT withdrawal (Fig 6C). Similar to the *sox9* expression profiles, *amh* and *amhr2* expression was significantly decreased after 4 days of MT withdrawal (Fig 6D and 6E). Thus, these results demonstrated that the maleness induced by chemical treatment was an unstable status, and the male-to-female sex change instantly occurred in MT-induced passive maleness after MT withdrawal.

### Amh signals in the dormant stage of the gonad during the transient phase of the reversible male-to-female sex change

**Fig 7:**
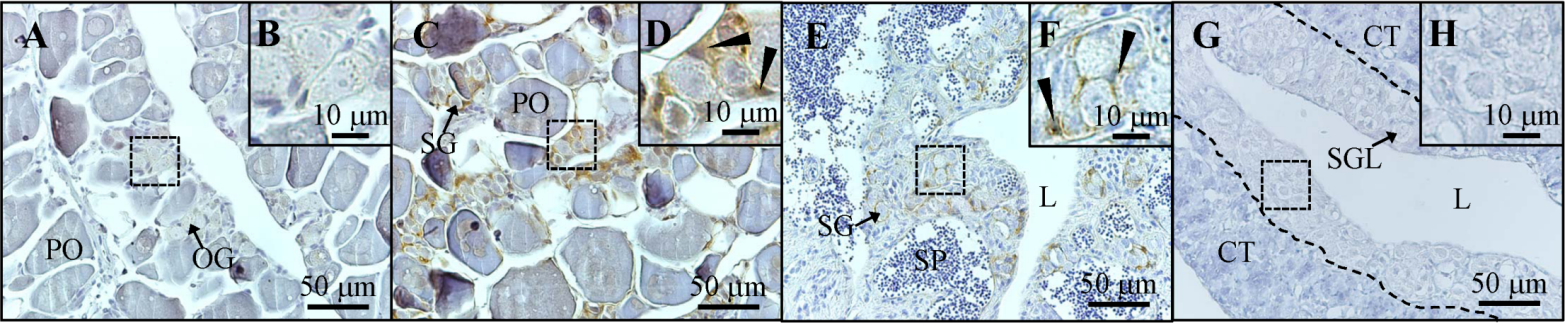
Immunohistochemical analysis of Amh in the gonad during reversible male-to-female sex change. Immunohistochemical staining of gonads at different stages using anti-Amh antiserum. (A and B) Primary oocyte stage in the female gonad. (C and D) Initial phase of the male-to-female reversible sex change in the gonad after a week of methyltestosterone (MT) administration. (E-F) Amh signals in the early stage (4 of 6 fish) of reversible male-to-female sex change after two weeks of MT withdrawal in MT-induced males (3 months treatment). (G-H) No Amh signals in the later stage (2 of 6 fish) of reversible male-to-female sex change after two weeks of MT withdrawal in MT-induced males (3 months treatment). The black arrowheads indicate the positive signal (brown color) of anti-Amh antiserum. CT, connective tissue; L, central lumen; PO, primary oocytes; OG, oogonia; SG, spermatogonia; SGL, type A spermatogonia-like cells.

According to the IHC staining of Amh, our results showed that Amh expression was absent in the control fish (Fig 7A and 7B). No signal of Amh was found in the pre-follicle cells of oogonia and early primary oocytes (Fig 7B). Amh signals were found in the pre-Sertoli cells of type A spermatogonia-like cells in the gonad after a week of MT administration (Fig 7C and 7D). After MT withdrawal in the MT-induced male (3 months treatment), advanced male germ cells showed increased proliferating activities (with BrdU-incorporation) in the early stage of male-to-female sex change [14]. In contrast, type A spermatogonia-like cells were in dormancy (without Brdu-incorporation) during male-to-female sex change [14]. Therefore, two stages (early stage and later stage) were divided in the transition phase of male-to-female sex change (Fig 7E and 7F; Fig 7G and 7H). In the early stage of the transition phase, there were many sperms with a small number of type A spermatogonia. In contrast, only type A spermatogonia-like cells were observed in the gonad in the later stage of the transition phase during male-to-female sex change. Amh signals were observed in the surrounding cells of type A spermatogonia-like cells in the early stage of transition phase gonad after two weeks of MT withdrawal (4 of 6 fish) during the male-to-female sex change (Fig 7E and 7F). However, in some fish (2 of 6 fish), Amh signals were not found in the later stage of transition phase gonad at the same period (after two weeks of MT withdrawal) (Fig 7G and 7H). This inconsistency may be due to the slight difference of gonadal stage. Thus, no Amh signals in the somatic cells surrounding the type-A spermatogonia like cells revealed these early germ cells may alter the fate from male to female.

## Discussion

This study was focused on how to identify the sexual fate during bi-directional sex change. We suggest here that Amh signaling should play a dual role in the male differentiation and regulates type A spermatogonia differentiation, and germ cells might maintain their numbers during reversible sex change through Amh signaling in the orange-spotted grouper.

### Gonadal differentiation in hermaphroditic fish

Unlike most gonochoristic vertebrates, sex determination is caused by an initial switch of testis-differentiating or an ovary-differentiating molecular cascade in an exclusive manner [1, 9]. In hermaphroditic fish, the sex was determined in the initial gonadal differentiation (primary sex determination) and was affected by the stability of the sexual phase (secondary sex determination) [32, 33]). The sexual phase decision for secondary sex determination in hermaphroditic fish is dependent on various parameters, including age, body size, and social factors [9]. However, a chemical-induced sex change is a transient status, and a reversible sex change was found after chemical administration was withdrawn in the present study and previous studies of the protogynous grouper [14, 27] and protandrous black porgy [12, 13]. Thus, hermaphroditic fish regulate both sexes at the same time; that is, one sex develops, and another sex regresses.

### Germ-soma interaction in fish

No molecular characteristics of both sexes were found in the dormant gonad during the transient phase from male to female function in the grouper [14]. In the fish, the sexual fate of early germ cells was flexible during development, becoming either type A spermatogonia or oogonia, and sexual fate was dependent on gonadal sex [34]. In XX genotype medaka, loss-of-*foxl3* function in the oogonia developed functional sperm in the expanded germinal epithelium of a functional ovary [35]. Moreover, ectopic oocytes were observed in the E2-indeced femaleness after E2 was withdrawn in black porgy [24, 36]. These results revealed that sexual fate determination in germ cells is not only regulated in an E2-dependent manner (with follicle cells) but is also regulated in an E2-independent manner (without follicle cells).

In the protogynous grouper [14] and protogynous wrasse [37], the oocyte-depleted follicle cells immediately alter the function of soma cells from female to male in the newly regenerated male gonad. Conversely, follicle cells are not derived from male soma cells during the reversible male-to-female sex change in grouper [14]. In black porgy, ectopic located oocytes in regenerated testes can alter the sexual fate of gonadal soma cells from male to female function [24, 36]. Furthermore, in medaka [38], zebrafish [39], and gibel carp [40], loss-of-germ cells results in the male characteristics of gonadal soma cells and phenotypic male. Taken together, female germ cells may play an important role in the maintenance of female soma function in grouper.

### Role of Amh signaling in testes

In the present study, we demonstrated that *amh* and *amhr2* expression was significantly increased in AI- and MT-induced female-to-male sex change (passive maleness). Our study also confirmed that Amh is localized in the Sertoli cells surrounding the spermatogonia. Conversely, low or absent expression of *amh*/Amh was found in femaleness (PO stage and VO stage) and transition phase of female-to-male sex change (RO stage). Thus, this male-specific Amh was not expressed in oocyte-depleted follicle cells during female-to-male sex change. Similarly, a male-specific *amh* expression pattern has been reported in some fish, and amh was expressed exclusively in the fish testes [17]. In medaka [41], zebrafish [23], and black porgy [24, 42], *amh* and its receptor *amhr2* were expressed in Sertoli cells in testes. In testicular tissue culture, Amh treatment inhibits the proliferation of type A spermatogonia in zebrafish [23] and black porgy [24]. Furthermore, loss of Amh signaling in the *hotei* mutant (*amhr2* mutant) medaka results in germ cell hyperproliferation [41]. Conversely, XY *hotei* mutant germ cells undergo type A spermatogonia-like division in the XY wild-type gonad [41]. Taken together, these results suggest that Amh is an important factor in Sertoli cells to influence the male differentiation of germ line cells in grouper.

### Amh plays an important role in germ line maintenance during reversible sex change

Our present and previous studies [14] demonstrated that chemical-induced maleness is rapidly reversed from male to female after chemical administration is withdrawn. High proliferating activity in advanced germ cells and dormancy in type A spermatogonia-like cells were shown in the beginning of reversible male-to-female sex change, and then advanced male germ cells were depleted [14]. In the present study, we demonstrated that male-related gene (*dmrt1* and *sox9*) expression was rapidly decreased at day 1 and day 4 of chemical termination, respectively. Conversely, no change was found in *cyp11b2* expression. In the grouper, *sox9* expression is a male marker, and Sox9 was only expressed in Sertoli cells [29]. In addition, grouper Dmrt1 protein exists only in spermatogonia, primary spermatocytes and secondary spermatocytes [29]. Moreover, the key enzyme for 11-KT, Cyp11b2, exhibited strong signals in the grouper interstitial cells [30]. Our data showed a low *sox9* expression and a stable *cyp11b2* expression in the gonadal tissue of MT withdrawal fish. The present study demonstrated that *amh* and *amhr2* expression was rapidly decreased after chemical termination. However, Amh showed a constant expression in the surrounding cells of type A spermatogonia-like cells. Thus, we suggest that the Amh expression pattern is related to the maintenance of the early germ cell stage, and the gonad remained at the dormant stage during the transient phase of reversible male-to-female sex change. Taken together, we suggest that Amh might regulate the differentiation of type A spermatogonia. This constant Amh expression in the surrounding cells of dormant type A spermatogonia-like cells might be related to the prevention of the advanced differentiation of gem cells during reversible male-to-female sex change in the grouper. We suggest this dormant type A spermatogonia-like cells might maintain the numbers of early germ cells for the future development of femaleness (for the transdifferentiation of oogonia from type A spermatogonia).

### Conclusions

We demonstrated that Amh was localized in Sertoli cells. Our data indicated that Amh was expressed in the surrounding cells of type A spermatogonia-like cells at the beginning of the female-to-male sex change. Our data also indicated that Amh was constantly expressed in the surrounding cells of type A spermatogonia-like cells at the transient phase of the reversible male-to-female sex change. Amh signaling is suggested to play roles in regulating male differentiation during female-to-male sex change and in preventing advanced development in type-A spermatogonia-like cell during the reversible male-to-female sex change.

## Supporting information

S1 Table Sexual phase (gonadal status, st.) and number of fish with body size during the experimental period including the process of female-to-male sex change in MT-implanted fish (methyltestosterone; 100 μg/mg pellet, 200 mg pellet/kg body weight). The sexual phase (status 1–7) was referred to the legend of Fig 1.
(DOCX)

S2 Table Sexual phase (gonadal status, st.) and number of fish with body size during the experimental period including AI/MT-induced female-to-male sex change and the male-to-female sex change in AI/MT-terminated fish (AI = aromatase inhibitor, MT = methyltestosterone). The sexual phase (status 3, 7 and 9) was referred to the legend of Fig 1.
(DOCX)

S1 Fig Schematic picture showing the experiments 1 and 2. (A) Experiment 1: To enhance the process of female-to-male sex change (status 5, status 6, and status 7), we conducted the sex change by applying MT (methyltestosterone; 100 μg MT/mg pellet, 200 mg pellet/kg of body weight, n = 18) implantation in > 2.5-yrs-old fish. Approximately all control fish (23/24) showed femaleness (status 3 and status 4) during the experimental period. (B) Experiment 2: To obtain fish in the different sexual phases of bi-directional sex change, we induced chemical-induced female-to-male sex change by feeding with an aromatase inhibitor (20 mg/kg of feed) and MT (50 mg/kg of feed) for 3 months. Reversible male-to-female sex change was observed after chemicals withdrawal. A dormant gonad (status 8) was a transient phase of male-to-female sex change after chemicals withdrawal. The white bar denotes the femaleness. The black bar denotes the chemical-induced maleness. The grey arrowhead shows the time of pellet (without MT) implantation in control fish. The white arrowhead shows the time of MT-implantation in the treated fish. The black arrowhead shows the time for sample collection.
(TIFF)

S2 Fig The deduced protein sequence of grouper Amh and it conserved structure from different fishes. Based on the alignment of protein sequence between orange-spotted grouper (Ec) and the other fish (black porgy, As; European sea bass, Dl; zebrafish, Dr), the conserved region are showed in different colors. Green color denotes the signal peptide. Red color denotes the predicted plasmin protease cleavage site (RXXR). Grey color denotes the predicted glycosylation site. Underline and bold letters shows the peptide fragment for immunized antibody.
(TIFF)

S3 Fig A phylogenetic tree comparing the amino acid sequences of Amhr2 and Tgfb-related receptor from different fish. Analysis of the phylogenetic relationship between Tgfb family-related tyrosine kinase receptor members showed that grouper Amhr2 clusters with other fish Amhr2 proteins and is not the closely related to other tyrosine kinase receptor members. The sequences were aligned by a multiple sequence alignment using MUSCLE. The phylogenetic tree was constructed using the neighbor-joining method. The number at each node represents the bootstrap probability (%). The red bar shows the grouper Amhr2. Acvr1, type 1 activin A receptor (also known as Alk-2, activin receptor-like kinase-2); Acvr1b, type 1b activin A receptor (also known as Alk-4, activin receptor-like kinase-4); Acvr1c, type 1c activin A receptor (also known as Alk-7, activin receptor-like kinase-7); Acvr2a, type 2a activin receptor; Acvr2b, type 2b activin receptor; Amhr2, type 2 Amh receptor; Bmpr1a, type 1a bone morphogenetic protein receptor; Bmpr1b, type 1b bone morphogenetic protein receptor; Bmpr2, type 2 bone morphogenetic protein receptor; Tgfbr1, type 1 transforming growth factor beta; Tgfbr2, type 2 transforming growth factor beta.
(TIFF)

S4 Fig Schematic figure of the Amh structure. (A) Hypothetical Amh structure. (B) Detected size of endogenous Amh using the WB (Western blot analysis). The arrowhead denotes the predicted plasmin protease cleavage site (RXXR) in fish. Red arrowhead and black arrowhead denotes the conserved site (RXXRXXR) and non-conserved site (RXXR) in fish, respectively.
(TIFF)
