# Supplementary material for: Expression profile of amh/Amh during bi-directional sex change in the protogynous orange-spotted grouper Epinephelus coioides
Source: PLoS One. 2017 Oct 10;12(10):e0185864. doi: 10.1371/journal.pone.0185864 (PMC5634590; doi:10.1371/journal.pone.0185864)
Supplement: S1 Table — The sexual phase (status 1–7) was referred to the legend of Fig 1. (DOCX) [file pone.0185864.s001.docx]

**S1 Table. Sexual phase (gonadal status, st.) and number of fish with body size during the experimental period including the process of female-to-male sex change in MT-implanted fish (methyltestosterone; 100 μg/mg pellet, 200 mg pellet/kg body weight). The sexual phase (status 1 - 7) was referred to the legend of Fig. 1.**

|  |  |  |  |  |  |  |
| --- | --- | --- | --- | --- | --- | --- |
|  |  |  |  |  | Sexual phase [status (No.)] |  |
| Administration periods (day) | Sample No. | Total length (cm) | Body weight (g) | Female | Interphase | Male |
| Initial control | 6 | 34.0 ± 1.1 | 507.1 ± 63.6 | st.3 (4) + st. 4 (2) | 0 | 0 |
| **Control** |  |  |  |  |  |  |
| 7 | 6 | 30.8 ± 0.4 | 346.3 ± 24.6 | st.3 (5) + st.4 (1) | 0 | 0 |
| 14 | 6 | 31.1 ± 0.6 | 344.1 ± 19.9 | st.3 (3) + st.4 (2) | 0 | st. 7 (1) |
| 28 | 6 | 31.5 ± 0.3 | 377.8 ± 31.3 | st.3 (4) + st.4 (2) | 0 | 0 |
| **MT** |  |  |  |  |  |  |
| 7 | 6 | 32.7 ± 0.7 | 385.2 ± 24.8 | st. 3 (1) + st.4 (1) | st. 5 (2) | st. 6 (2) |
| 14 | 6 | 33.7 ± 1.3 | 491.5 ± 53.4 | st.4 (2) | st. 5 (3) | st. 6 (1) |
| 28 | 6 | 32.9 ± 0.4 | 409.5 ± 18.1 | st. 4 (1) | st. 5 (3) | st. 6 (1) + st. 7 (1) |
