# Supplementary material for: Expression profile of amh/Amh during bi-directional sex change in the protogynous orange-spotted grouper Epinephelus coioides
Source: PLoS One. 2017 Oct 10;12(10):e0185864. doi: 10.1371/journal.pone.0185864 (PMC5634590; doi:10.1371/journal.pone.0185864)
Supplement: S2 Table — The sexual phase (status 3, 7 and 9) was referred to the legend of Fig 1. (DOCX) [file pone.0185864.s002.docx]

**S2 Table. Sexual phase (gonadal status, st.) and number of fish with body size during the experimental period including AI/MT-induced female-to-male sex change and the male-to-female sex change in AI/MT-terminated fish (AI = aromatase inhibitor, MT = methyltestosterone). The sexual phase (status 3, 7 and 9) was referred to the legend of Fig. 1.**

|  |  |  |  |  |  |  |
| --- | --- | --- | --- | --- | --- | --- |
| Administration periods |  |  |  |  | Sexual phase [status (No.)] |  |
| [treatment (months) + termination] | Sample No. | Total length (cm) | Body weight (g) | Female | Interphase | Male |
| Initial control | 12 | 25.5 ± 0.58 | 280.0 ± 21.11 | st. 3(12) | 0 | 0 |
| **Control** |  |  |  |  |  |  |
| 3 + 0 | 8 | 26.9 ± 1.5 | 367.4 ± 45.5 | st. 3 (8) | 0 | 0 |
| 3 + 3 | 3 | 28.0 ± 1.0 | 311.9 ± 41.7 | st. 3 (8) | 0 | 0 |
| 3 + 11 | 3 | 31.0 ± 2.1 | 440.7 ± 53.2 | st. 3 (8) | 0 | 0 |
| **AI** (20 mg/kg of diet) |  |  |  |  |  |  |
| 3 + 0 | 8 | 27.3 ± 0.6 | 316.5 ± 24.5 | 0 | 0 | st. 7 (8) |
| 3 + 3 | 3 | 28.3 ± 0.9 | 359.2 ± 34.8 | 0 | st. 8 (2) | st. 7 (1) |
| 3 + 11 | 3 | 34.0 ± 1.5 | 603.3 ± 16.9 | st. 3 (8) | 0 | 0 |
| **MT** (50 mg/kg of diet) |  |  |  |  |  |  |
| 3 + 0 | 8 | 27.4 ± 1.1 | 324.5 ± 34.8 | 0 | 0 | st. 7 (8) |
| 3 + 3 | 3 | 28.3 ± 1.5 | 359.6 ± 55.7 | 0 | st. 8 (2) | st. 7 (1) |
| 3 + 11 | 3 | 29.7 ± 0.3 | 490.0 ± 30.2 | st. 3 (8) | 0 | 0 |
