# Supplementary figures and images for: Expression profile of amh/Amh during bi-directional sex change in the protogynous orange-spotted grouper Epinephelus coioides
Source: PLoS One. 2017 Oct 10;12(10):e0185864. doi: 10.1371/journal.pone.0185864 (PMC5634590; doi:10.1371/journal.pone.0185864)

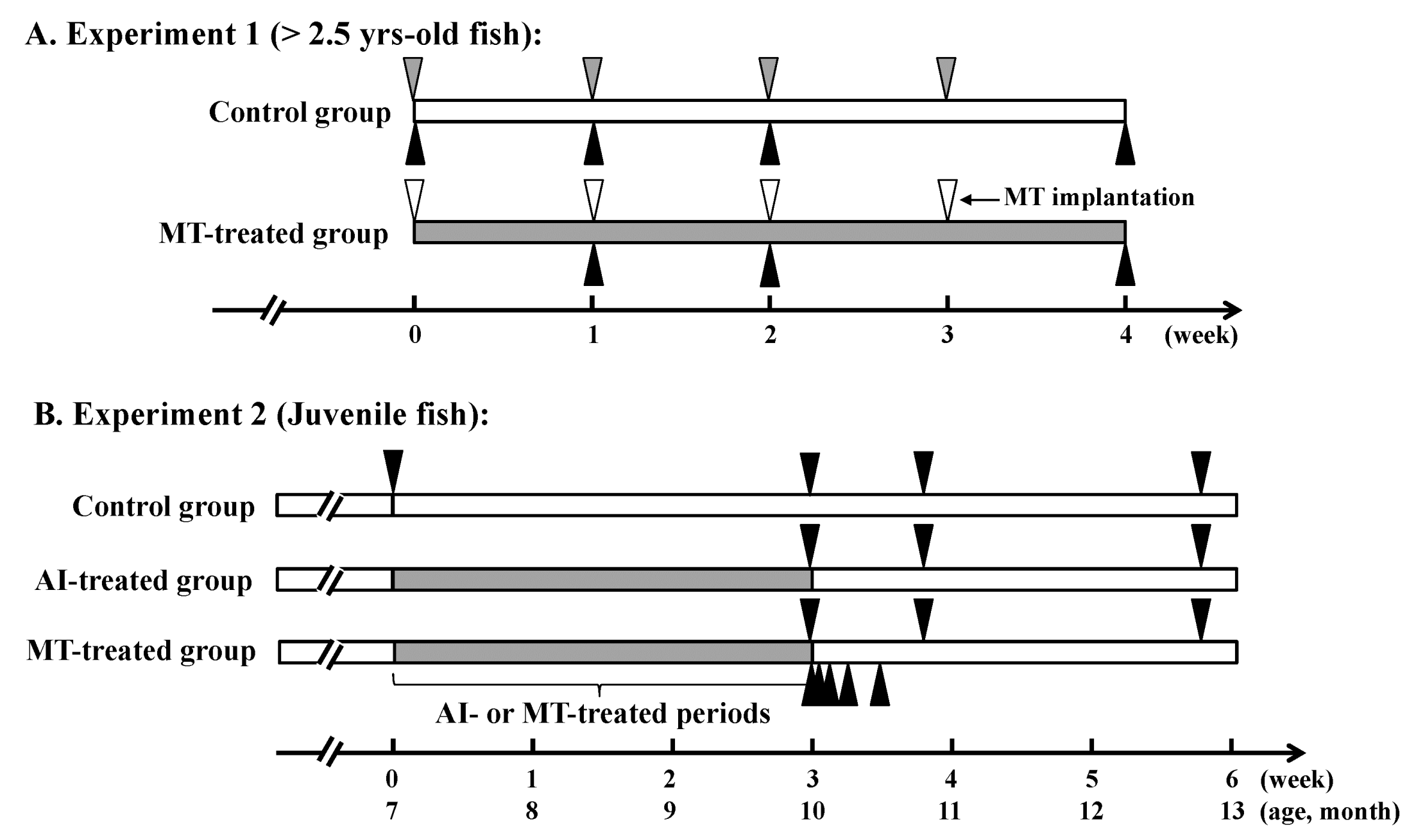

Supplement: S1 Fig — (A) Experiment 1: To enhance the process of female-to-male sex change (status 5, status 6, and status 7), we conducted the sex change by applying MT (methyltestosterone; 100 μg MT/mg pellet, 200 mg pellet/kg of body weight, n = 18) implantation in > 2.5-yrs-old fish. Approximately all control fish (23/24) showed femaleness (status 3 and status 4) during the experimental period. (B) Experiment 2: To obtain fish in the different sexual phases of bi-directional sex change, we induced chemical-induced female-to-male sex change by feeding with an aromatase inhibitor (20 mg/kg of feed) and MT (50 mg/kg of feed) for 3 months. Reversible male-to-female sex change was observed after chemicals withdrawal. A dormant gonad (status 8) was a transient phase of male-to-female sex change after chemicals withdrawal. The white bar denotes the femaleness. The black bar denotes the chemical-induced maleness. The grey arrowhead shows the time of pellet (without MT) implantation in control fish. The white arrowhead shows the time of MT-implantation in the treated fish. The black arrowhead shows the time for sample collection. (TIFF) [file pone.0185864.s003.tiff]

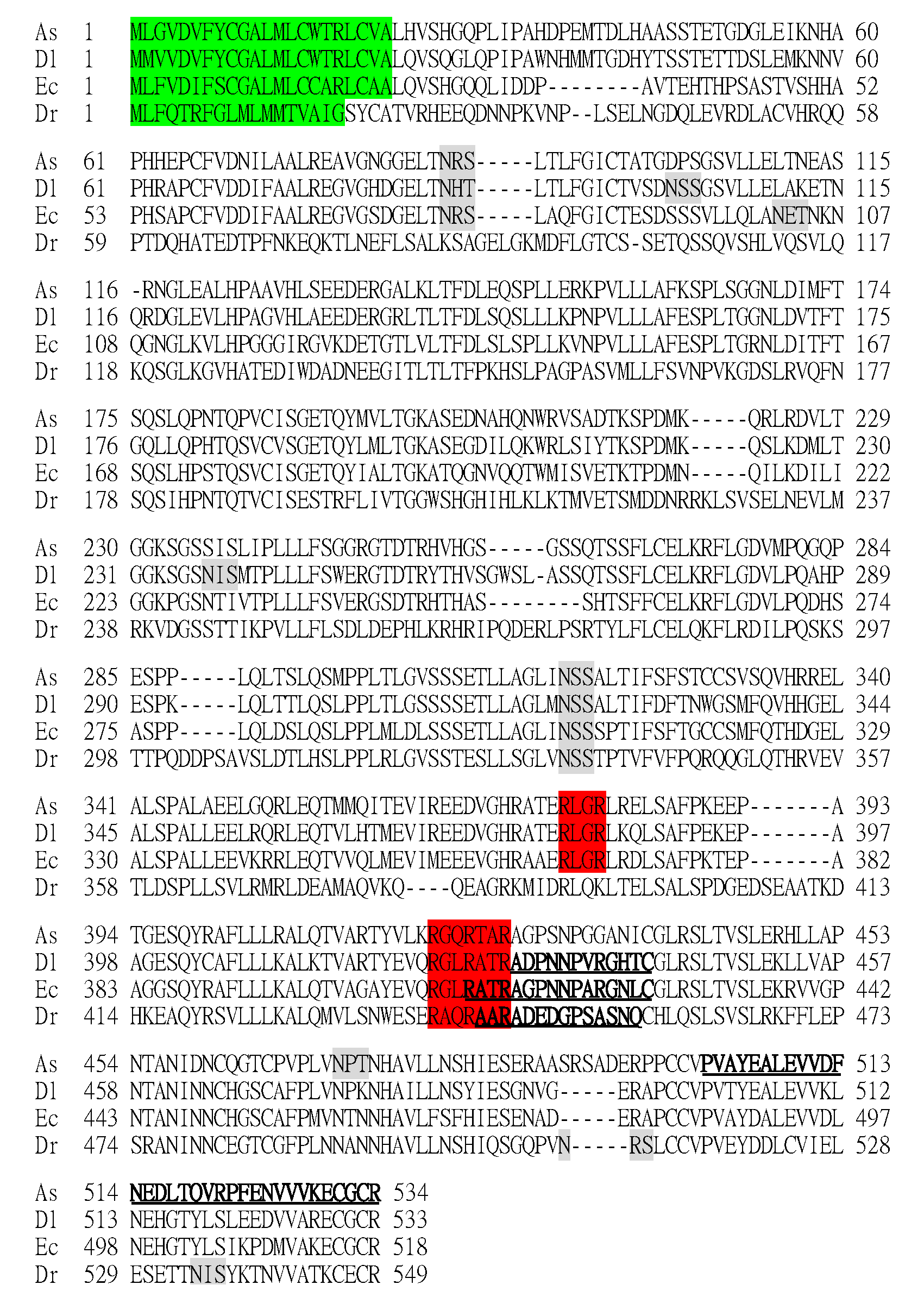

Supplement: S2 Fig — Based on the alignment of protein sequence between orange-spotted grouper (Ec) and the other fish (black porgy, As; European sea bass, Dl; zebrafish, Dr), the conserved region are showed in different colors. Green color denotes the signal peptide. Red color denotes the predicted plasmin protease cleavage site (RXXR). Grey color denotes the predicted glycosylation site. Underline and bold letters shows the peptide fragment for immunized antibody. (TIFF) [file pone.0185864.s004.tiff]

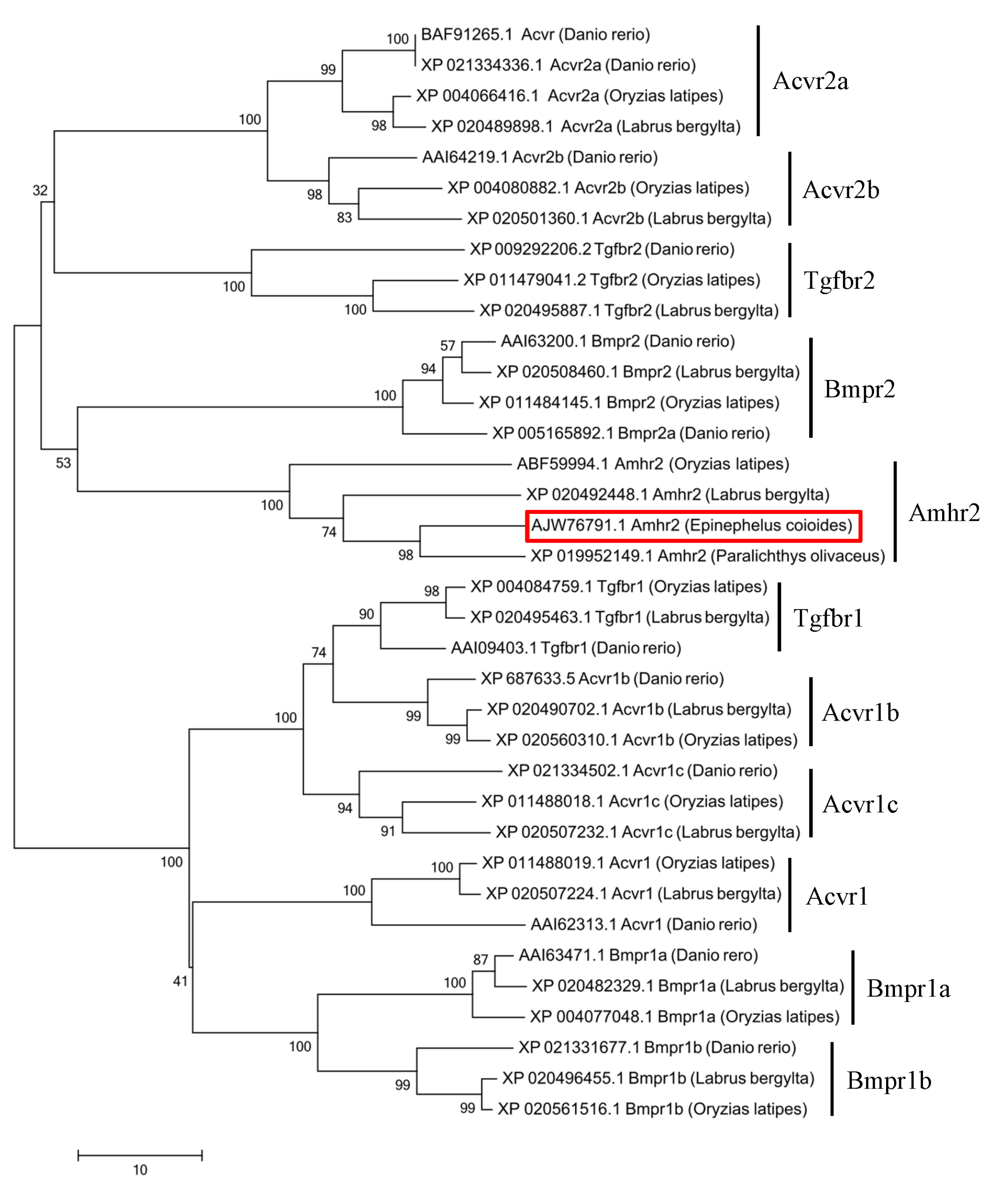

Supplement: S3 Fig — Analysis of the phylogenetic relationship between Tgfb family-related tyrosine kinase receptor members showed that grouper Amhr2 clusters with other fish Amhr2 proteins and is not the closely related to other tyrosine kinase receptor members. The sequences were aligned by a multiple sequence alignment using MUSCLE. The phylogenetic tree was constructed using the neighbor-joining method. The number at each node represents the bootstrap probability (%). The red bar shows the grouper Amhr2. Acvr1, type 1 activin A receptor (also known as Alk-2, activin receptor-like kinase-2); Acvr1b, type 1b activin A receptor (also known as Alk-4, activin receptor-like kinase-4); Acvr1c, type 1c activin A receptor (also known as Alk-7, activin receptor-like kinase-7); Acvr2a, type 2a activin receptor; Acvr2b, type 2b activin receptor; Amhr2, type 2 Amh receptor; Bmpr1a, type 1a bone morphogenetic protein receptor; Bmpr1b, type 1b bone morphogenetic protein receptor; Bmpr2, type 2 bone morphogenetic protein receptor; Tgfbr1, type 1 transforming growth factor beta; Tgfbr2, type 2 transforming growth factor beta. (TIFF) [file pone.0185864.s005.tiff]

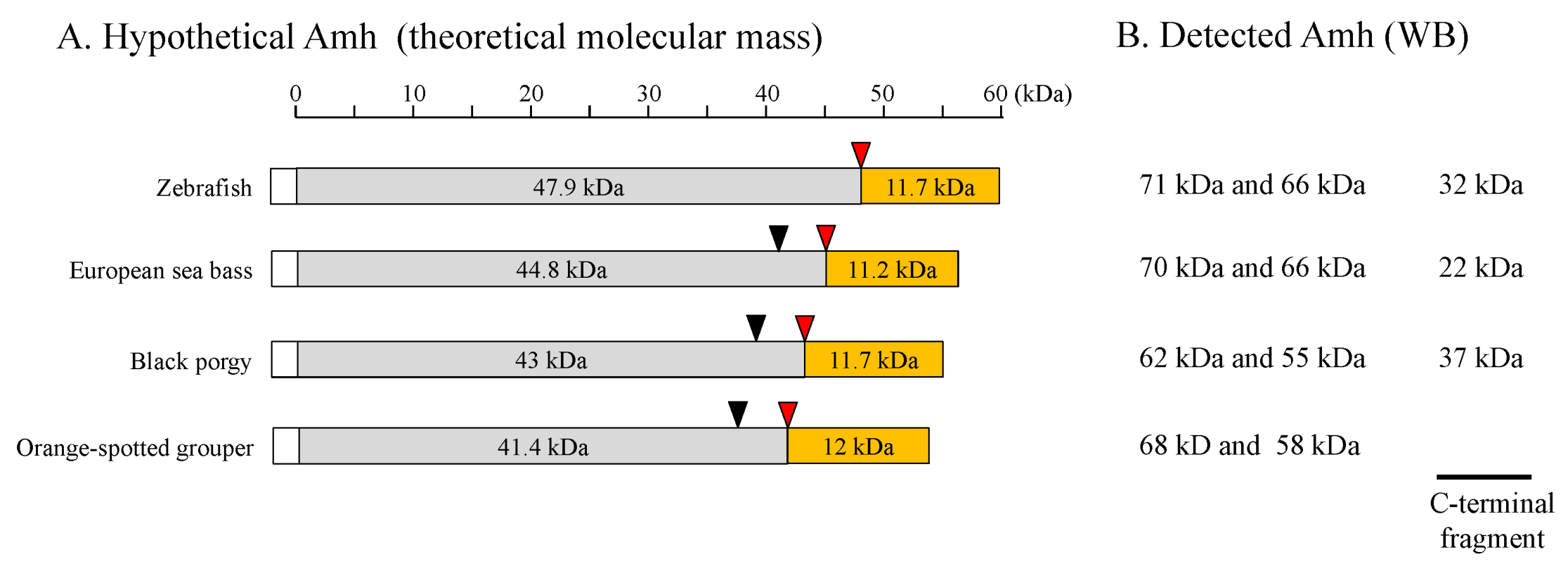

Supplement: S4 Fig — (A) Hypothetical Amh structure. (B) Detected size of endogenous Amh using the WB (Western blot analysis). The arrowhead denotes the predicted plasmin protease cleavage site (RXXR) in fish. Red arrowhead and black arrowhead denotes the conserved site (RXXRXXR) and non-conserved site (RXXR) in fish, respectively. (TIFF) [file pone.0185864.s006.tiff]
